# The pharmacological effects and safety of the raw and prepared folium of *Epimedium brevicornu* Maxim. on improving kidney-yang deficiency syndrome and sexual dysfunction

**DOI:** 10.3389/fphar.2023.1233468

**Published:** 2023-07-14

**Authors:** Kai Wang, Juntao Li, Xinyu Zheng, Jian Xu, Zhe Wang, Senjie Li, Qiang Yang, Yue Wu, Dong-Hua Yang, Shen Yao, Xiangwei Zheng

**Affiliations:** ^1^ Science and Technology Experiment Center, Shanghai University of Traditional Chinese Medicine, Shanghai, China; ^2^ Engineering Research Center of Modern Preparation Technology of Traditional Chinese Medicine, Ministry of Education, Innovation Research Institute of Traditional Chinese Medicine, Shanghai University of Traditional Chinese Medicine, Shanghai, China; ^3^ Hubei Provincial Key Laboratory of Quality and Safety of Traditional Chinese Medicine Health Food, Jing Brand Research Institute, Jing Brand Co., Ltd., Daye, Hubei, China; ^4^ New York College of Traditional Chinese Medicine, Mineola, NY, United States; ^5^ Sanlin Community Health Service Center of Shanghai Pudong New District, Shanghai, China

**Keywords:** processed folium of *Epimedium brevicornu* Maxim, raw folium of *Epimedium brevicornu* Maxim, hypothalamus-pituitary-adrenal axis, sexual dysfunction, neuroendocrine-immune network

## Abstract

**Background:** Kidney-Yang deficiency syndrome (KDS) is a group of diseases related to hypothalamic-pituitary-adrenal (HPA) axis and sexual dysfunction. The folium of *Epimedium brevicornu* Maxim. (FEB) includes raw and prepared slices, named RFEB and PFEB, respectively. PFEB is traditionally believed to be good for tonifying kidney-Yang and improving sexual dysfunction. However, there are few studies comparing the pharmacological effects of RFEB and PFEB, and their underlying mechanisms. In this study, we aimed to compare the effects and safety of RFEB and PFEB on the HPA axis and sexual function. Additionally, the mechanisms of their roles in relation to the neuroendocrine-immune (NEI) network in the KDS model mice were explored.

**Methods:** Male adult C57BL/6 mice were treated with corticosterone to establish a KDS mouse model, and RFEB and PFEB were administered intragastrically. Corticotropin releasing hormone (CRH), adrenocorticotropic hormone (ACTH), cyclic adenosine monophosphate (cAMP), cyclic guanosine monophosphate (cGMP), testosterone levels and oxidative damage indexes were measured. The mRNA and protein levels of *CRH* and *ACTH* in hypothalamus and pituitary, endothelial nitric oxide synthase (eNOS) and phosphodiesterase 5 (PDE5) in corpus cavernosum were examined. TNFα, IL-6, NF-κB, eNOS and PDE5 were investigated in mouse corpus cavernosum.

**Results:** Our results showed that PFEB was more effective than RFEB in increasing corticosterone-suppressed *ACTH* levels, enhancing *CRH* levels and cAMP/cGMP ratio, and reducing oxidative damage. *In vivo*, PFEB significantly increased eNOS and inhibited PDE5 expression in corpus cavernosum. PFEB showed stronger protective effect on normal spleen lymphocytes from apoptosis both *in vitro* and *in vivo*. Additionally, it noticeably inhibited the levels of inflammatory cytokines in corpus cavernosum. Both RFEB and PFEB were safe and did not cause any clinical signs of toxicity in mice at the dosage of 20 times dosages of that in the Chinese Pharmacopeia.

**Conclusion:** We demonstrated that PFEB was better than RFEB at tonifying the kidney-Yang by comparing their effects on improving the NEI network, which includes the HPA axis, immune system and corpus cavernosum. This study revealed that PFEB could significantly improve the sexual function of KDS mice by regulating the HPA axis and activating the immune system through the NEI network.

## 1 Introduction

In Traditional Chinese medicine (TCM), the “kidney-Yang” governs growth, development, and aging of human body. Clinical symptoms of kidney-Yang deficiency commonly include sexual dysfunction, waist and knees soreness, tinnitus and deafness, tooth loss, and others. Modern medical research shows that patients with kidney-Yang deficiency syndrome (KDS) are frequently accompanied with different diseases related to dysfunction of hypothalamic-pituitary-adrenal (HPA) axis and sexual organs (Shen, 2012). Previous pathological and pharmacological studies have demonstrated that KDS is closely associated with the HPA axis and the regulating center located in hypothalamus could be the target organ of kidney-Yang tonifying (Shen, 2012). It was also found that herbs tonifying the kidney-Yang are involved in neuroendocrine-immune (NEI) network and could activate immune system, and promote secretion of sex hormone and growth hormone (Shen, 2005; Shen, 2012; Huang et al., 2013b). Therefore, KDS animal model can be established by intake a large-dose of glucocorticoids (GCs) to induce HPA axis disorders (Shen, 2012; Dai et al., 2017).

Epimedii Folium (EF), known as Yinyanghuo in Chinese, has been commonly used as a TCM over the last two thousand of years for the treatment of various diseases. The *Epimedium genus* includes at least 68 species that exist as herbs, present in land around the Mediterranean and east Asia, and there are approximately 58 species in China (Xu et al., 2020). Various medicinal materials and preparations of Yinyanghuo as Chinese medicine and dietary supplements are traded to Japan, South Korea, Europe and America from China.

The folium of *Epimedium brevicornu* Maxim. (FEB) from a variety of Yinyanghuo, was recorded in the 2020 edition of Chinese Pharmacopoeia. And it is increasingly valued by pharmacognosticians because of its abundant wild resources and relatively stable quality of medicinal components (Xu et al., 2008). By TCM theory, the prepared slice of FEB (PFEB) is beneficial in reinforcing the kidney-Yang whereas the raw products (RFEB) have the advantage of strengthening tendons and bones. Thus, the PFEB is commonly used to treat sexual dysfunction, and RFES is used for treatment of osteoporosis. However, there are few studies comparing their effect on HPA axis and the sexual function of KDS animal model. Moreover, it is not clear how the FEB regulation of HPA axis links to the restoration of sexual function in the entire NEI network.

There were only two experimental studies reported the protective effects of FEB on HPA axis in KDS rat model (Huang et al., 2013a; An et al., 2015): The extract of FEB is beneficial in treating hydrocortisone-induced-KDS rats through improving HPA axis and endocrine system, which involved in releasing corticotropin releasing hormone from hypothalamus, producing serum adrenocorticotropic hormone in anterior pituitary, and secreting corticosteroids from adrenal cortex. FEB flavonoids affect GCs-induced-HPA axis-suppression by upregulation of *ACTH* and insulin-like growth factor II. Additionally, there are a few literature reports on how Yinyanghuo improves sexual function in the experimental animals: It was found that PFEB significantly promoted the growth and endocrine function of the testicular tissues in normal rat (Niu, 1989). The water and alcohol extracts of both RFEB and PFEB could significantly inhibit the atrophy of accessory organs of castrated mice (Wang et al., 2005). Preliminary clinical trials had shown that FEB granules could effectively improve the sperm number and motility in male infertility patients with abnormal semen (Gu et al., 2018). Chiu et al. (2006) revealed that the mechanism on FEB treatment on Erectile Dysfunction (ED) is involved in relaxing the corpus cavernosum smooth muscle through activation of NO/cGMP/PDE5 signaling pathway.

At present, there was no systematic study on the mechanism of prepared slices of EF on regulating HPA axis and immune system in the NEI network in KDS animal model. As part of the series of research on the mechanism of action of the processed products of Yinyanghuo, this study aimed to compare the difference of RFEB and PFEB in the effects and safety involving HPA axis, immune system and sexual function in KDS mice. Additionally, the internal connection of these systems will be discuss. This study will provide rationale for the application of Yinyanghuo.

## 2 Materials and methods

### 2.1. Plant sources

The folium of *E. brevicorn*. was purchased and identified by Dr. *Jian Xu* in Jing Brand Co., Ltd., Hubei Province, China, in May 2019. The specimen vouchers (20190502) had been stored in the Hubei Provincial Key Laboratory for Quality and Safety of Traditional Chinese Medicine.

### 2.2 Preparation of the RFEB and PFEB extract

According to Chinese Pharmacopoeia, processing procedures of the folium of *E. brevicorn* include eliminating foreign matter, pick the leaves, spray with water, soften slightly, cut into slivers and dried. Stir-bake the slivers of FEB with refined suet by gentle heating until an evenlustre is produced, then remove and cool the slivers. Using 20 kg of refined suet per 100 kg of FEB, the PFEB (5 kg) were extracted with 50% EtOH (the solvent multiples were 8 times and 7 times in turn, 1.5 h, each time) at 83°C and with 95% EtOH at 78°C. RFEB were extracted by the same method without suet. Then RFEB and PFEB were concentrated in the rotary evaporator, eventually dried into powder in the vacuum drying box, and their extraction rate was 7.5%.

### 2.3 Dectection of the total flavonoid content in RFEB and PFEB

According to the detection method for the total flavonoid content of Yinyanghuo in the 2020 edition of the Chinese Pharmacopoeia, The detection steps were as follows:

Preparation of reference solution: Icariin, accurately weighed, added with methanol to produce 0.1 mg per 1 mL. The solution is used as the mother liquor for backup.

Preparation of sample solution: Accurately weigh 0.2 g of RFEB and PFEB dry extract powder and place them in a 50 mL volumetric flask. Add an appropriate amount of methanol to completely dissolve and replenish it to the mark. Shake well and serve as the test solution.

### 2.4 Animal grouping and sample collection

Forty eight-week-old C57BL/6 mice were acquired from Charles River (Beijing, China) and kept in cages with room temperature of 22°C–25°C and 70% humidity. Animal welfare and experimental procedures were followed in accordance with the institutional guidelines for the care and use of laboratory animals and related ethical regulations of Shanghai University of Traditional Chinese Medicine (2022011). The mice were divided into the control group, KDS group, RFEB group, and PFEB group, 10 mice in each group. The control group was subcutaneously injected with 0.2 mL of normal saline (NS) each day, while the other three groups were subcutaneously injected with 20 mg/kg of corticosterone in 1% dimethyl sulfoxide (DMSO) each day. Based on the pharmaceutical results achieved in the preliminary experiment, as well as the human clinical dosage of 9 g of the folium of *E. brevicorn* per 70 kg body weight (China Pharmacopoeia Committee, 2015), mice in RFEB group and PFEB group were correspondingly gavaged with a dosage of 2.25 g extract of RFEB and PFEB per kg body weight daily, equating 2 times of the human clinical dosage, for consecutive 21 days. (The two kinds of extract are about 1.486 g crude drug per milliliter). The control group and KDS group were given distilled water of the same volume.

On 8:00 a.m. of the 22nd day, the mice were weighed and anesthetized. Blood samples were collected from the fundus venous plexus, and blood serum was isolated. The cavernous body was removed and divided into two even parts. One-half was fixed with 4% paraformaldehyde solution, while the other half was frozen with liquid nitrogen. The thymus gland and the spleen were weighed and calculated for organ coefficient.

Organ Coefficient (mg/g) = Organ Weight (mg)/Body Weight (g)

### 2.5 Serum hormonal indicators

By ELISA, the serum samples were tested for *ACTH* (Cusabio Technology, Wuhan, China) and testosterone (Elabscience, Wuhan, China) levels, according to the procedures given in the manufacturer’s manuals. The serum samples were tested for cAMP and cGMP levels using the cAMP GsHiRange kit and the cGMP HTRF kit (PerkinElmer, United States), and then the cAMP/cGMP ratios were calculated. Following blood biochemistry analysis, the serum samples were tested for superoxide dismutase (SOD) and MDA levels (Jiancheng, Nanjing, China.)

### 2.6 qRT-PCR

The hypothalamus, pituitary, and cavernous body tissues were individually ground in liquid nitrogen and lysed with cell lysis buffer. The total RNA was extracted using Trizol and tested for purity, and then reverse transcribed into cDNA using Super RT cDNA Synthesis Kit (Cwbio, Beijing, China). The cDNA was then amplified using the Ultra SYBR One Step RT-qPCR Kit (Cwbio). See Table 1 for the primer sequences. The amplification conditions were 95°C for 2 min, 38 cycles of 95°C for 30 s and 60°C for 30 s and finally 74°C for 30 s. The mRNA levels of eNOS, PDE5A1, *ACTH*, and *CRH* were calculated using the 2^−△△Ct^ method.

**TABLE 1:** List of primer sequences.

Name	Primer sequence
*CRH*-F1	ACC​TTC​TGC​GGG​AAG​TCT​TG
*CRH*-R1	TTT​TGG​CCA​AGC​GCA​ACA​TT
*ACTH*-R1	CGT​ACT​TCC​GGG​GGT​TTT​CA
*ACTH*-F1	GGC​TTG​CAA​ACT​CGA​CCT​CT
eNOS-F	GCA​CAT​TTG​GCA​ATG​GGG​AT
eNOS-R	CGG​GCC​TGA​CAT​TTC​CAT​GA
PDE5A1-F	CAACTCCGTGCGGTCG
PDE5A1-R	TCT​CTG​AAA​ACC​ATG​CGT​TGA
GAPDH-F1	GAG​TCA​ACG​GAT​TTG​GTC​GT
GAPDH-R1	TTG​ATT​TTG​GAG​GGA​TCT​CG

### 2.7 Western blot

The hypothalamus, pituitary, and cavernous body tissues were individually ground, homogenized using RIPA lysis buffer. The homogenates were rested on ice for 30 min, and immediately subjected to centrifugation with 12,000 × g in 4°C for 15 min, then the supernatant was collected. The total protein concentration in supernatant was determined according to the BCA Protein Assay Kit (Cwbio). 50 µg of total protein was separated using SDS-PAGE. The electrophoresis peptides were transferred to the PVDF membranes. The PVDF membranes were then blocked for 2 h using 5% nonfat dried milk and probed with antibodies at 4°C. After washing 3 times using TBST, the PVDF membranes were incubated for 2 h using horseradish peroxidase-conjugated secondary antibodies, and then ECL (a chemiluminescence agent) was used to develop the protein bands. The signal intensity was analyzed using Gel-Pro Analyzer 4.0 software (Media Cybernetics, Silver spring, MD, United States). The GAPDH was used as an internal control to assess the protein loading equivalence. The eNOS antibodies (1:500,ab300071), PDE5A1 antibodies (1:500, ab259945), *ACTH* antibodies (1:500, ab32893), *CRH* antibodies (1:500, ab184238), and GAPDH antibodies were purchased from Abcam.

### 2.8 PDE5A1 inhibition assays

This test adopted the method we have published previously (Li et al., 2022). Briefly, all samples were diluted in assay buffer which contained 100 mM MgCl_2_ and 50 mM Tris-HCl (pH 8.0). 30μL PDE5A1 solution (0.0625 μg/mL, ab56614 Abcam) and 30 uL extraction solution were mixed at room temperature and incubated for 5 min, followed by addition of 30 μL cGMP solution (7.5 μg/mL, G6129, Sigma), and the reaction was carried out at 35°C for 90 min. The PDE5A1 inhibitors were diluted to 7 concentration series with a; 2-fold gradient. After the reaction, each vial was incubated in 100°C boiling water for 5 min to inactivate PDE5A1 activity, and then cooled to be tested with HPLC. Percentage inhibition was calculated using the peak area of HPLC. Two individual operations were performed on different dates as the repetition.

The half maximal inhibitory concentration (IC50) of each extraction solution towards PDE5A1 was evaluated.

### 2.9 Isolation of splenic lymphocytes and apoptosis analysis

The mouse spleen was isolated aseptically, and minced into small pieces and filtered through 100-um nylon mesh to acquire the cell suspension. Splenic lymphocytes were isolated after centrifugation at 250 *g* for 10 min and cell fractionation on lymphocyte separation medium (density = 1.081). After the white blood cell layer was collected and washed, the cells were cultured in RPMI-1640 medium containing 10% Fetal bovine serum (FBS) for 2 h, then the non-adherent cells (splenic lymphocytes) were harvested and adjusted to 1 × 106 cells/mL in the culture medium.

Splenic lymphocytes were labeled with Annexin V and Propridium Iodide (PI), and left to react at room temperature for 30 min in the dark. The apoptosis rate was detected by fluorescence-activated cell sorting (FACS) analysis.

### 2.10 CCK-8 assay on splenic lymphocytes proliferation

The splenic lymphocytes were adjusted to a concentration of 2 × 10^6^/mL, and 100 μL was added to each well in a 96-well culture plate. Concanavalin A (Con A) of 3 μg/mL and the extract of different concentrations were added. The lymphocytes were cultured for 72 h in 5% CO_2_ at 37°C. The cell viability was measured using Cell Counting Kit-8 (CCK-8) method.

### 2.11 Immunohistochemistry analysis

The paraformaldehyde-fixed cavernous body was dehydrated, embedded in paraffin, and cut into 5-um thick sections. The sections were deparaffinized, hydrated, incubated for 10 min in 3% H_2_O_2_ at room temperature to stop endogenous peroxidase activity. The sections were blocked for 30 min in 10% goat blood serum, and then incubated in a specific primary antibody solution overnight at 4°C. Next, the sections were incubated in a biotinylated secondary antibody solution at room temperature for 30 min, then incubated in a streptavidin-horseradish peroxidase complex diluted by 1:200 at room temperature for 30 min. Finally, using DAB solution (3,3′-diaminobenzidine tetrahydrochloride solution), the sections were observed under microscope for color development. Finally, the sections were counterstained with hematoxylin, dehydrated, and mounted with resin. The eNOS antibody (ab300071), PDE5A1 antibody (ab259945), TNF-α antibody (ab1793), IL-6 antibody (ab290735), and NF-κB antibody (ab32536) were purchased from Abcam.

### 2.12 Toxicity evaluation

Mice (five mice per group) were orally administered with the FEB extract at 22.5 g/kg BW every day for 2 weeks. The mice in control group were given distilled water. Mortality, and body weight were monitored. The major organs, such as heart, liver, stomach, and kidneys were collected for evaluations after 14 days. Blood samples were subjected to centrifugation (10 min at 1,400 g) for serum separation. Biochemical assay was used to assess alanine aminotransferase (ALT), aspartate aminotransferase (AST) and lactate dehydrogenase (LDH) activity determination, and urea and creatinine concentration, by clinical analyzer (HITACHI 7180, Japan).

### 2.13 Statistical processing

Statistical software SPSS 17.0 was used for analysis, and the data were expressed as mean ± standard deviation (x ± sd). One-way ANOVA and LSD-t test were used for comparison between multiple groups, with a significant difference defined at *p* < 0.05.

## 3 Results

### 3.1 Flavonoid content in RFEB and PFEB

Take the mother liquor under “*2.3*” and place it in a 10 mL volumetric flask. Add methanol to prepare solutions with concentrations of 0.0055, 0.0073, 0.011, 0.0165, and 0.022 mg/mL, respectively. With methanol as the blank, refer to Ultraviolet–visible spectroscopy (Appendix VA in the 2020 edition of the Chinese Pharmacopoeia), measure the absorbance at the wavelength of 270 nm, take the concentration of the reference solution as the abscissa, and the absorbance value as the ordinate, to obtain the linear regression equation: y = 52.425x -0.0649 (r = 0.9997, linear range 0.0052 ∼ 0.026 mg/mL). Based on this, the total flavonoid contents of RFEB and PFEB were measured as follows: 4.98, 5.40 (%) (*n* = 3).

### 3.2 The effect of FEB extracts on KDS treatment

**FIGURE 1:**
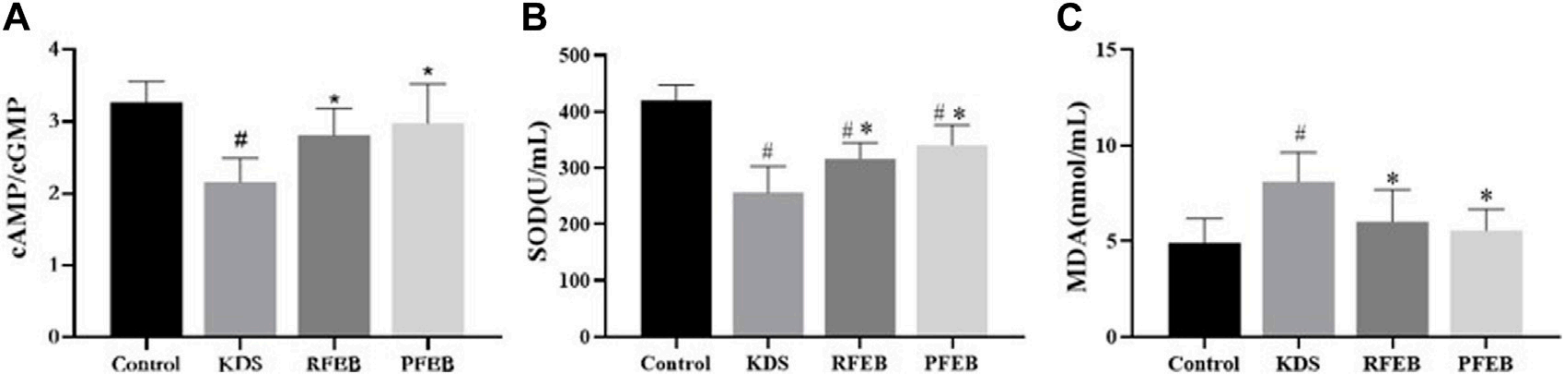
Effects of RFEB and PFEB on the changes in the second messengers and oxidative stress molecules in KDS mice. **(A)** Serum cAMP/cGMP Tatio. **(B)**Serum S0D-activity. **(C)** Serum MDA content. Mean ± SD (*n* = 10/group), #*p* < 0.05 vs. control; *p < 0.05 vs. KDS, ANOVA, LSD-t-test.

After treatment with RFEB or PFEB, some changes were observed in the KDS mice. Figure 1 showed the changes in the biochemical indices in rat serum. Cyclin AMP is the second messenger with the strongest effect among those induced by *ACTH* in the HPA axis, which affects downstream signaling pathways. Cyclin GMP is another second messenger in the HPA axis. Since cAMP and cGMP have been shown to link to the Yin-Yang theory and the suppression of cAMP/cGMP ratio was shown to be a manifestation of Yang deficiency, we evaluated the ratio of cAMP/cGMP in serum (Goldberg et al., 1975; Brian et al., 1990; Jin et al., 2020). SOD and MDA are indicators reflecting oxidative stress levels. Corticosterone caused cAMP/cGMP ratio (Figure 1A) and SOD level (Figure 1B) to decrease, and MDA level to increase (Figure 1C); while RFEB and PFEB both raised cAMP/cGMP ratio and SOD level, and decreased MDA level. Both cAMP/cGMP ratio and MDA levels returned to normal range after treatment.

### 3.3 The comparative effect of RFEB and PFEB on hormone secretion in the HPA axis of KDS mice

**FIGURE 2:**
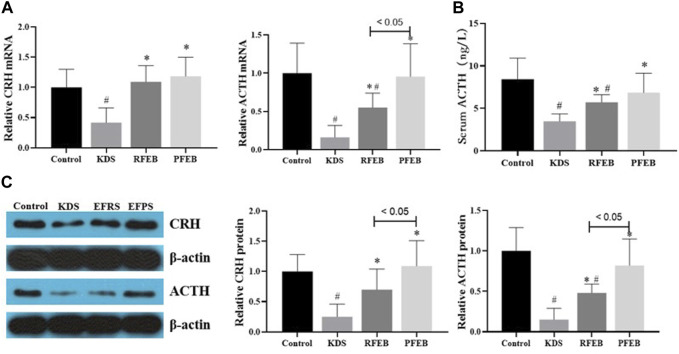
Effect of RFEB and FEB on hormone secretion inthe HPA axis of KDS mice. **(A)** CRH mRNA.levels in the hypothalamus and ACTH RNA in thepituitary. **(B)** Serum ACTH content. **(C)** CRH protein levels in the hypothalamus and ACTH protein levels in the pituitary. Mean ± SD (*n* = 10/group), #*p* < 0.05 vs. control; *p < 0.05 vs. KDS, ANOVA, LSD-t test.

As Figure 2 showed, KDS mice showed a decrease in both *CRH* levels in the hypothalamus, as well as *ACTH* levels in the pituitary and serum, compared to control mice (*p* < 0.05). After RFEB and PFEB treatment, both mRNA and protein levels of *CRH* and *ACTH* markedly increased, and the treatment with PFEB increased *CRH* and *ACTH* mRNA by PFEB to the similar level of control group. Compared to the RFEB treatment group, the *ACTH* mRNA and protein levels in the pituitary of PFEB treatment group increased with a statistically significant difference (*p* < 0.05).

### 3.4 The comparative effect of RFEB and PFEB on the corpus cavernosum of KDS mice

**FIGURE 3:**
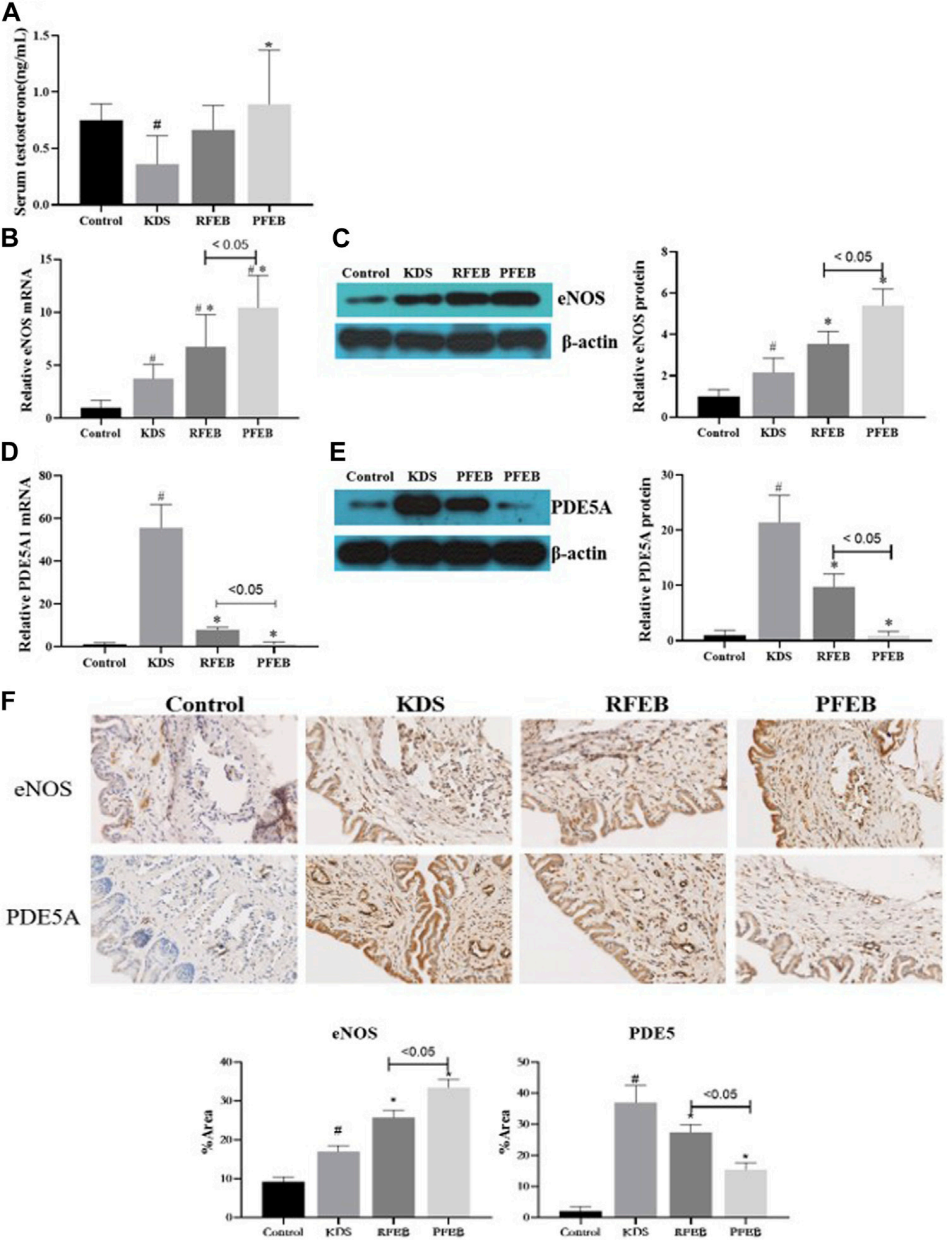
Effect of RFEB and PFEB affect the expression of sex hormones, eNOS and PDE5A in the KDS mice. **(A)** Serum testosterone content. eNOS mRNA **(B)** and protein levels **(C)** incorpus cavernosum. PDE5A mRNA **(D)** and protein levels **(E)**. **(F)** incorpus cavernosum. eNOS and PDE5A expression incorpus cavernosum by immunohistochemistry. Mean ± SD (*n* = 10/group), #*p* < 0.05 vs. control; *p < 0.05 vs. KDS, ANOVA, LSD-t test.

Testosterone, eNOS and PDE5 are important indicators reflecting ED symptom. As shown in Figure 3A, the serum testosterone level in KDS mice was significantly lower than that of control mice (*p* < 0.05), whereas mice treated with PFEB had testosterone level significantly higher than KDS mice (*p* < 0.05). Compared to control group, the mRNA and protein levels of eNOS and PDE5A1 of corpus cavernosum increased in KDS mice. (*p* < 0.05). As shown in Figures 3B–E, with the RFEB and PFEB treatment, the expression of eNOS in corpus cavernosum showed progressive increase, with PFEB group having higher levels than RFEB group (*p* < 0.05). Figures 3D, E showed that RFEB and PFEB treatment markedly decrease PDE5A expression which was raised by corticosterone, and PDE5A returned to normal range by PFEB treatment. Figure 3F showed the eNOS and PDE5A expression in corpus cavernosum by immunohistochemistry, which showed similar change.

### 3.5 Comparisons of the inhibitory potency of FEB extracts on PDE5A1 in vitro

**FIGURE 4:**
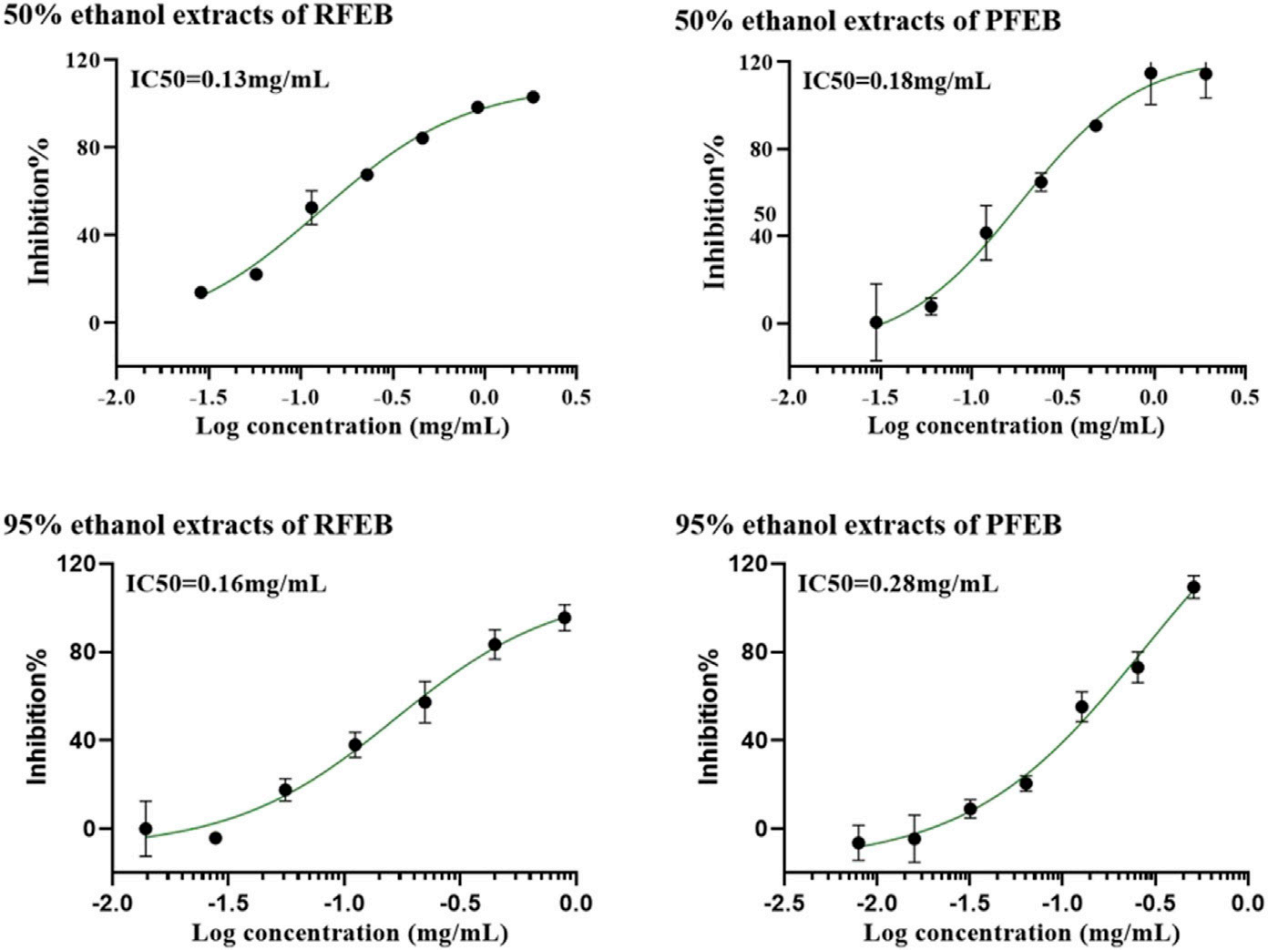
*In vitro* IC_50_ curve of various EF extract of PDE5A1.

Figure 4 showed the 50% inhibitive concentration (IC50) of different types of FEB extracts on PDE5A1 activity. Both the RFEB and the PFEB extract, using 50% and 95% ethanol, inhibited PDE5A1 activity. The four types of extracts have IC50s between 0.13 mg/mL - 0.28 mg/mL, and there was no significant difference in their inhibiting potency on PDE5A1 *in vitro*.

### 3.6 The comparative effect of FEB extracts on KDS mouse immune cell response *in vivo* and *in vitro*

**FIGURE 5:**
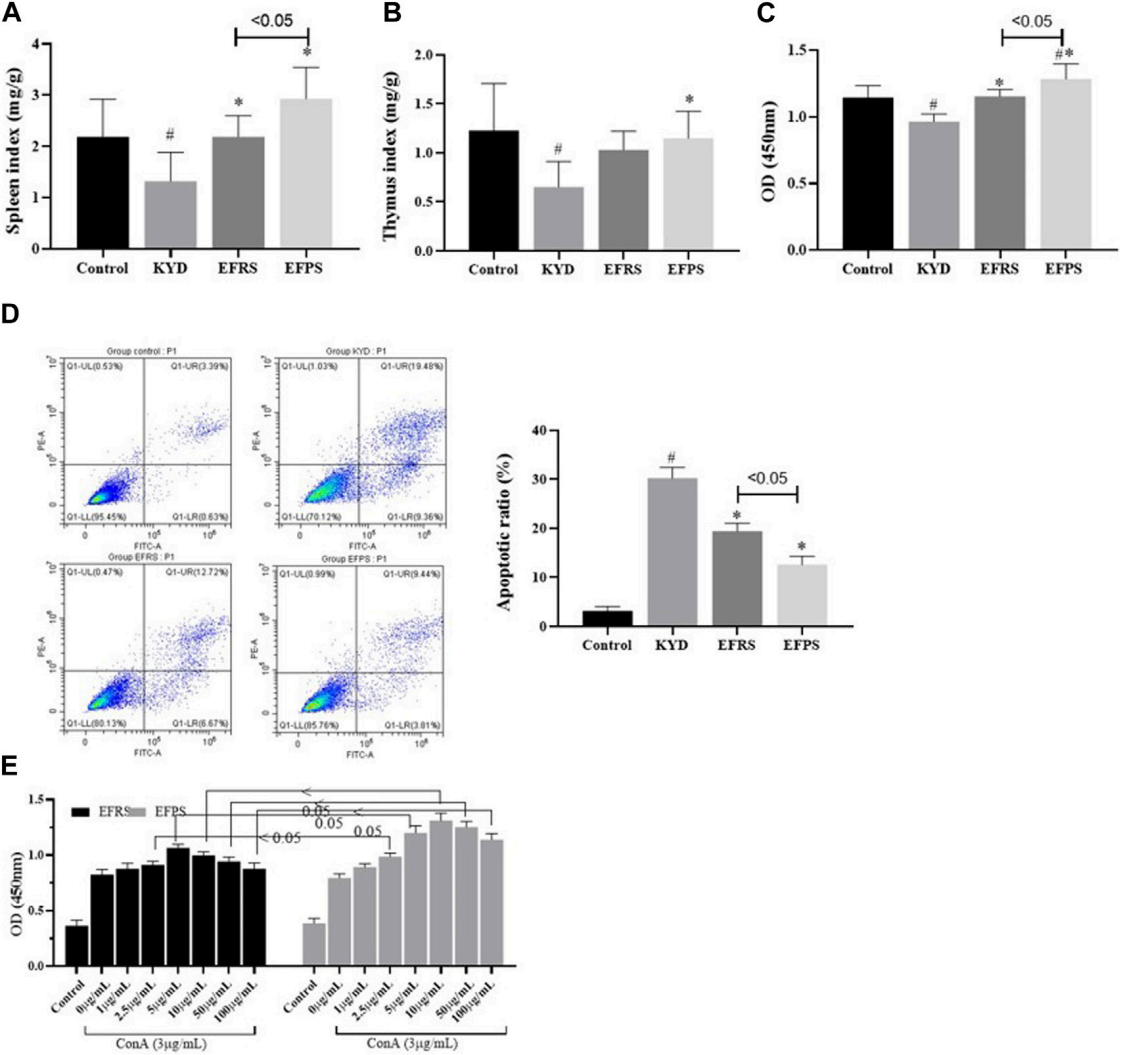
Effect of RFEB and PFEB on KDS mouse immune response. Spleen index **(A)** and thymus index **(B)** (*n* = 0/group). The proliferation **(C)** and apoptosis **(D)** of spleen lymphocytes of various groups *in vitro*. (*n* = 5/group). **(E)** The proliferation of normal lymphocytes (*n* = 5/group). Mean ± SD# *p* < 0.05 vs. control; *p* < 0.05 vs. KDS, ANOVA, LSD-t test.

As shown in Figures 5A, B, the spleen index and thymus index of KDS mice were noticeably lower than those of control mice (*p* < 0.05). RFEB and PFEB significantly attenuated spleen and thymus atrophy induced by corticosterone, indicated by the increases of spleen index and thymus index (*p* < 0.05). Concanavalin A (Con A) acts as a mitogen, stimulating the receptors on the lymphocyte membrane and inducing lymphocyte proliferation and immune response. The level of lymphocyte proliferation can reflect the strength of the body’s immune response; therefore, in the present study this indicator was used to examine the effect of RFEB and PFEB on immune regulation. Figures 5C, D showed the proliferation and apoptosis of spleen lymphocytes *in vitro*. Compared to the lymphocytes of control mice, the *in vitro* lymphocyte proliferation of KDS mice was markedly low, while apoptosis was more significant. RFEB and PFEB increased lymphocyte proliferation and decreased apoptosis to different degrees, and PFEB showed better performance than RFEB. The effect of RFEB and PFEB on normal lymphocyte proliferation *in vitro* was compared and shown in Figure 5E, after 48 h of cell culture, compared to the lymphocytes of control mice, Con A noticeably increased cell proliferation (*p* < 0.05). When RFEB concentration was below 5 ug/mL, or when PFEB concentration was below 10 ug/mL, as concentration increases, OD value gradually increased. When dosage was higher than 2.5 μg/mL, PFEB showed better performance in stimulating lymphocyte proliferation compared to RFEB with the same concentration (*p* < 0.05). When concentration of RFEB was higher than 5 ug/mL, or when PFEB was higher than 10 ug/mL, OD value decreased as concentration increased. Microscopic observation showed no significant change in morphology. There was no sign of cytoplastic shrinkage or other kinds of cytotoxicity. Therefore, the decrease in lymphocyte proliferation should not be attributed to cytotoxicity.

### 3.7 The comparative effect of RFEB and PFEB on inflammatory cytokines in corpus cavernosum of KDS mice

**FIGURE 6:**
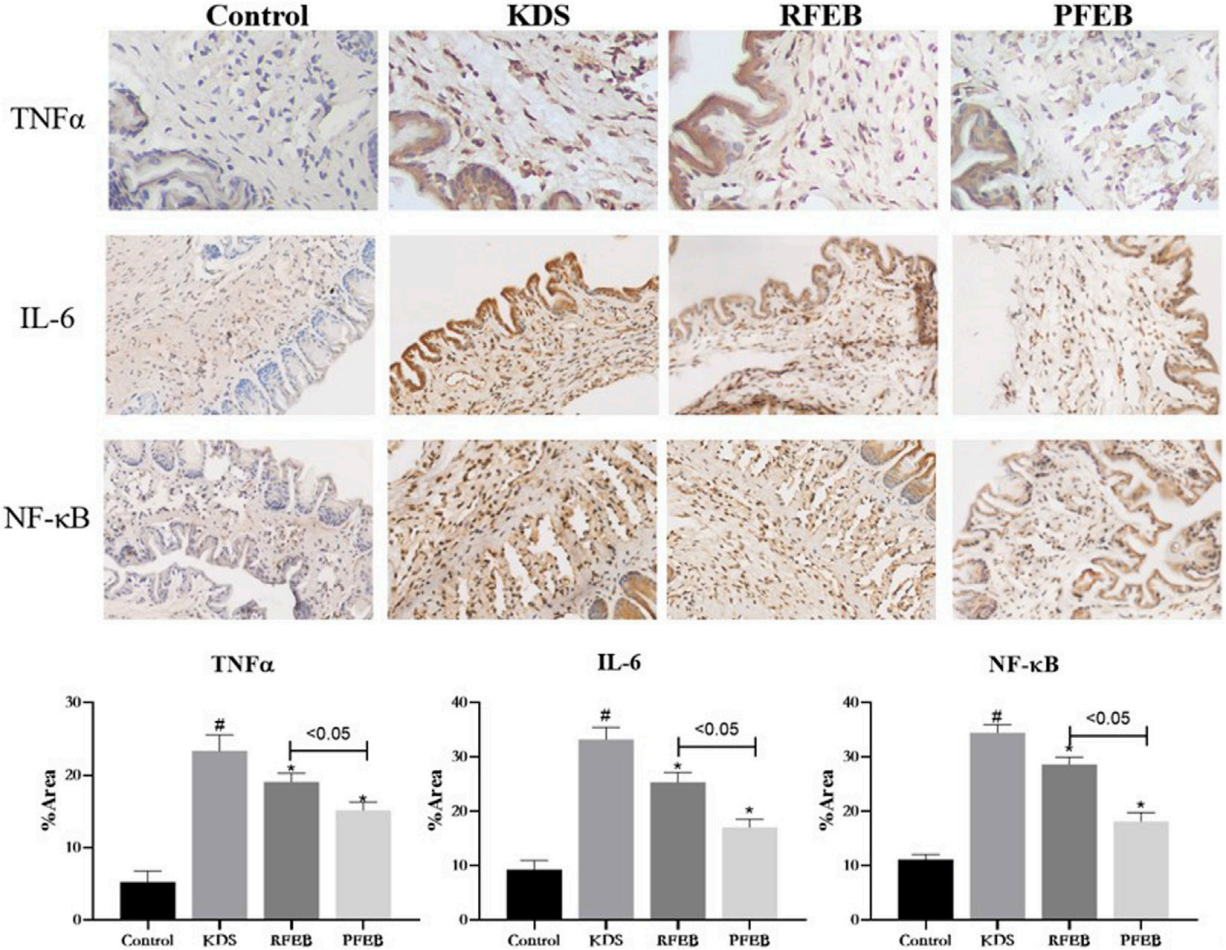
Effect of RFEB and IFEB on TNFα IL-6 and NF-κB expression in-corpus cavernosum of mice. Mean ± SD < (*n* = 10 group), #*p* < 0.05 control; vs. KDS, ANOVA, LSD-t test.

As Figure 6 showed, the inflammation in corpus cavernosum were observed. Both RFEB and PFEB inhibited the up-regulated inflammatory cytokines (TNFα, IL-6 and NF-κB) in corpus cavernosum of KDS mice (*p* < 0.05), and the down-regulated level of PFEB treatment was more obvious than that of RFEB (*p* <0.05).

### 3.8 Toxicity evaluation

**FIGURE 7:**
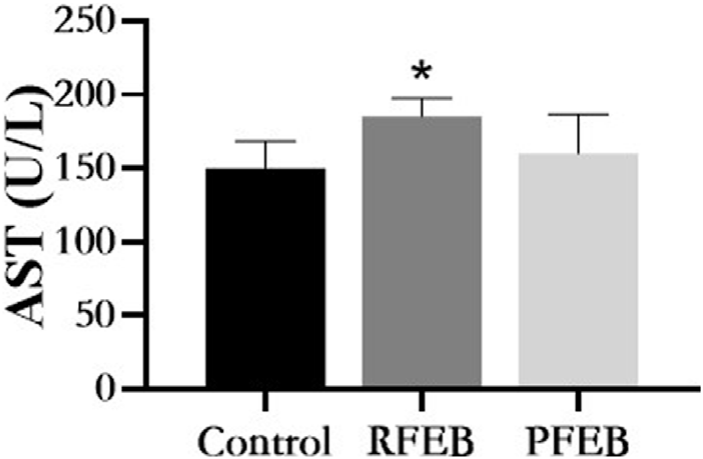
Effect of RFEB and PFEB on serum AST of mice. Mean ± SD (*n* = 5/group), *p < 0.05 vs., control, ANSOVA, LSD t-test.

All the mice remained normal under FEB treatment at the dosage of 20 times the human clinical dosage, with no any adverse behavioral changes. Animals treated with PFEB or RFEB showed weight gain equivalent to control mice. No changes in organ coefficient, morphology, or color were observed during analysis of the heart, liver, stomach, and kidneys (data not shown). Except for AST, all serum parameters (ALT, urea, creatinine, LDH) were in normal range and there was no difference between the groups. AST treated with RFEB (185.0 ± 12.4 U/L) were higher than control (150.5 ± 18.3U/L) and PFEB (160.1 ± 26.4U/L) group (Figure 7).

## 4 Discussion

### 4.1 FEB improve KDS via NEI network

After almost half of a century’s research, Zi-yin Shen suggested that the pathogenesis of KDS in TCM was associated with HPA axis, and the organs involved in the adrenal gland, gonads, and thyroidgland (Shen, 2005). The *ACTH*, cortisol (CORT) and cAMP level in Yang deficiency subjects declined significantly in comparison to healthy ones (Wang et al., 2008; Hu et al., 2018). Through the systematical analysis of the relationship between kidney-Yang deficiency and TCM clinical symptoms and related genes, it is found that the gene *CRH* is closely related to KDS. TCM clinical studies have confirmed that *Epimedium* flavonoids can reconstruct the balance of NEI network by increasing serum *ACTH* levels (Zha, 1982), recovering gonadal function and serum cortisol (Hua et al., 2003; Thomas et al., 2008; Xiong et al., 2009), as well as regulating cell apoptosis and proliferation in KDS patients (Xiao et al., 2000; Lin et al., 2002; Jin et al., 2010; Shen, 2012; Gong et al., 2015; Tan et al., 2017).

The Kidney-tonifying treatment based on the theory of the “HPA axis” has explained its underlying mechanism in treating kidney-Yang deficiency diseases and obtained clinical effectiveness, and KDS may have the potential relationship with ED (Gong et al., 2015). However, no study has reported the systemic organ changes and mechanism of TCM in tonifying kidney-Yang and the treatment of ED, which makes this research of important value for healthcare guidance and clinical application.

#### 4.1.1 Deregulation of endocrine and immune function, and sexual dysfunction co-exist in KDS

In this study, subcutaneous injection of corticosterone was used to disrupt HPA axis in KDS mice. The apparent decrease of serum cAMP/cGMP ratio and increase of oxidative stress markers confirmed the presence of KDS. Decreased levels of *CRH* in the hypothalamus and *ACTH* in the pituitary and serum indicated damage to the HPA axis. Decreased spleen index and thymus index indicated that kidney-Yang deficiency and HPA damage caused the atrophy of immune organs. In addition, the increased expression levels of eNOS and PDE5 in the corpus cavernosum and the decreased serum testosterone indicated that they also caused dysfunction of sexual organs. These data suggested that the KDS mouse model was successfully established, and their endocrine secretion, immune and sexual functions were impaired.

#### 4.1.2 FEB improves the neuroendocrine system of HPA axis and KDS index

Clinical studies have found that *Epimedium* extract increases *ACTH* and corticosterone levels in the plasma of patients who took prednisone (Cai et al., 1998). This present study found that FEB could not only increase corticosterone-suppressed *ACTH* levels *in vivo*, but also enhance *CRH* levels and cAMP/cGMP ratio, and reduce oxidative damage. This study was the first to compare the effects of RFEB and PFEB on the improvement of HPA endocrine secretion and KDS. PFEB showed a stronger effect than RFEB on the improvement of *CRH* levels, cAMP/cGMP ratio and oxidative damage at the indicated doses. PFEB was more effective than RFEB in improving *ACTH* levels.

#### 4.1.3 FEB reduces eNOS uncoupling and inhibit PDE5

In the concept of TCM, patients with HPA axis disorders have ED symptoms (Citron et al., 1996; Salvio et al., 2021). Moreover, in TCM clinical practice, sexual dysfunction is one of the typical symptoms of kidney-Yang deficiency. This study confirmed that FEB increased the level of eNOS induced by HPA axis disorders, and the effect of PFEB was significantly better than that of RFEB. It has been reported that eNOS coupling means normal electron transfer and function, while uncoupled eNOS under pathological conditions results in a decrease in NO, an increase in superoxide anion (O^2−^), and aggravation of oxidative stress, resulting in endothelial dysfunction (Scmidt and Alp, 2007; Perlins et al., 2012). For example, interleukin-1β (IL-1β) stimulus and noise exposure cause the uncoupling and dysregulation of eNOS by over-activating the HPA axis, which in turn leads to increased ROS and vascular dysfunction (Gadek-Michalska et al., 2012; Daiberab et al., 2020). In this study, the HPA axis disorders increased the eNOS level and serum oxidative stress biomarkers in the corpus cavernosum, however, FEB treatment reduced ROS and further increased eNOS expression. eNOS uncoupling might explain the relationship between the two changes. It was speculated that as FEB treatment increased eNOS expression and simultaneously reduced eNOS uncoupling, then to enhance NO, and therefore reduced oxidative stress. Further experiments are required to confirm this speculation. The result confirmed that the HPA axis disorders in KDS caused ED-like changes in the first messenger of the corpus cavernosum, and FEB had a protective effect against ED.

In addition, FEB reduced the level of PDE5 *in vivo*. PDE5 is highly expressed in the corpus cavernosum and has become a noteworthy target for drug screening in ED. Sildenafil (Viagra) is a representative drug for the treatment of ED. In this study, the *in vitro* inhibitive activities against PDE5 were similar to that using RFEB and PFEB usage, and interestingly, the *in vivo* inhibition on PDE5 expression by PFEB was better than that by RFEB. This *in vivo* pharmacological effect results are similar to the clinicalefficacy of PFEB in treating ED. It indicates that the different anti-ED effect between RFEB and PFEB was determined not by their enzyme inhibitory activity but by other mechanisms.

#### 4.1.4 FEB improve the immune system of KDS mice

The neuroendocrine system and the immune system are essential components that regulate the physiological activities of mammals, and they play important roles in the regulation of metabolism and fighting against infection and disease. There are dynamic and interactive transfer of signaling molecules between the two systems, which coordinate their activities. It is well known that glucocorticoids can inhibit the immune response of the body. If the HPA axis is severely dysfunctional, endocrine homeostasis and glucocorticoids released from the adrenal cortex will be disturbed, which in turn affects the immune system of the body and increases disease susceptibility of target organs.

In this study, it was found that FEB effectively increased the thymus index and spleen index of KDS mice and reduced the ratio of apoptotic lymphocytes in the spleen, and the protective effect of PFEB was stronger than that of RFEB. The results of the *in vitro* and *in vivo* experiments were consistent. Compared with RFEB, PFEB had a stronger effect on protecting immune organs and cells. In addition, the levels of inflammation-related cytokines TNFα, IL-6, and NF-κB in the corpus cavernosum were inhibited by FEB, especially PFEB. Similar results have been reported that *Epimedium* flavonoids significantly upregulate levels of FSH, sex hormones and lymphocytes amount in aged rats by secretion of GnRH, which is a powerful information transmission factor in NEI network (Huang et al., 2013a; An et al., 2015). Thus, in this study, it was speculated that FEB could act on the corpus cavernosum by regulating the HPA axis of the KDS animal model and activating the immune system through the signaling pathway of NEI network (Shen et al., 2004). The strong protective effect of PFEB on the NEI network might be the underlying mechanism that it was more efficient than RFEB in the treatment of kidney-Yang deficiency.

### 4.2 The composition changes of PFEB and RFEB, and the future prospects

The characteristic chemical components of *Epimedium* are 8-isopentenyl flavonoids in that icariin shows its protective effects on hydrocortisone-induced-KDS mice by regulating the HPA axis and endocrine system. Icariin also exhibit an IC_50_ value of 5.9 μM against PDE5A1 *in vitro* (Mario et al., 2008). It was worth noting that there were a few reports on the qualitative and quantitative analysis for the composition changes in the prepared slices of Yinyanghuo compared to raw products. The content of aglycones and secondary glycosides of flavonoids in Yinyanghuo increased due to the desugarization reactions in frying (Jing et al., 2009), and it was confirmed by some studies that the content of epimedin A, B and C decreased, while icariin increased when the herb was processed (Xie et al., 2011; Chen and Luo, 2013; Zhang et al., 2018; XU et al., 2021). And the increased icariin was proved to be a hydrolysis product of 3´´´-carbonyl-2´´-β-L-quinovosyl icariin instead of epimedin A, B and C (Sun et al., 2018). In addition, two (Sun et al., 2014), (Li et al., 2014) literature reported the compound formation mechanism in the processing of Yinyanghuo: During the processing, the increase of secondary glycosides and the formation of self-assembled micelles could be attributed to the absorption of isopentenyl flavonoid glycosides, which leads to the enhancement of warming kidney-Yang. The literature generally confirmed that the raw and processed Yinyanghuo showed the difference of substance components, which is the reason for the difference in their action effects and pharmacological mechanisms. However, a great deal of composition analysis should be researched and enriched for FEB, especially for PFEB. In addition, for the exploration of pharmacological mechanism, further efforts should be paid on signaling pathways and the connecting molecules among the neuroendocrine system, immune system, and target organs. These studies are necessary to reveal the mechanism of their different pharmacological effects. The current research could not fully explain the pharmacological effect and mechanism of FEB against KDS. Therefore, further study on its complex pharmacological effects, especially after processing, is warranted.

## 5 Conclusion

By comparing the pharmacological effects and mechanism of RFEB and PFEB on kidney-Yang deficiency mice, this study found that PFEB was more effective than RFEB in improving the HPA axis function, reducing symptoms of kidney-Yang deficiency, enhancing functions of gonadal and corpus cavernosum, and maintaining balance of immune system. Moreover, no toxicity was observed, suggesting the safety of RFEB and PFEB for clinical use.

## Figures and Tables

**Table:** 

**ACTH**	adrenocorticotropic hormone
**CAMP**	cyclic adenosine monophosphate
**CCK-8**	cell Counting Kit-8
**CGMP**	cyclic guanosine monophosphate
**ConA**	concanavalin A
**CRH**	corticotropin-releasing hormone
**DMSO**	dimethyl sulfoxide
**EF**	epimedii Folium
**eNOS**	endothelin nictric oxide synthase
**FACS**	fluorescence-activated cell sorting
**FEB**	the folium of Epimedium brevicornu Maxim.
**FSB**	fetal bovine serum
**GADPH**	glyceraldehyde-3-phosphate dehydrogenase
**GCs**	glucocorticoids
**HPA**	hypothalamic-pituitary-adrenal
**HPLC**	high performance liquid chromatography
**IC50**	half maximal inhibitory concentration
**IL-6**	interleukin-6
**KDS**	kidney-Yang deficiency syndrome
**MDA**	malondialdehyde
**NEI**	neuroendocrine-immune
**NF-κB**	nuclear factor kappa-B
**NO**	nitric oxide
**NS**	normal saline
**PDE 5**	phosphodiesterase type-5
**PFEB**	prepared slice of the folium of Epimedium brevicornu Maxim.
**PI**	propridium Iodide
**RFEB**	raw slices of the folium of Epimedium brevicornu Maxim.
**SOD**	superoxide dismutase
**TCM**	traditional Chinese medicine
**TNF-α**	tumor necrosis factor-α

## Data Availability

The original contributions presented in the study are included in the article/Supplementary Materials, further inquiries can be directed to the corresponding authors.
